# Efficacy and Safety of Direct-Acting Oral Anticoagulants Versus Warfarin in Patients With Mechanical Valve Replacement

**DOI:** 10.7759/cureus.84236

**Published:** 2025-05-16

**Authors:** Ryan J Browne, Niall Hill

**Affiliations:** 1 Cardiology, Cardiff University, Cardiff, GBR; 2 School of Medicine, Cardiff University, Cardiff, GBR

**Keywords:** acute ischemic stroke, anticoagulant therapy, cardiovascular outcomes trials, direct oral anticoagulants (doac), mechanical valve replacement, surgical aortic valve replacement (savr), warfarin therapy

## Abstract

This systematic review and meta-analysis aimed to compare the safety and efficacy of direct-acting oral anticoagulants (DOACs) and warfarin in patients with mechanical heart valves, specifically assessing the risks of ischemic stroke, thromboembolism, and major hemorrhage. We included randomized controlled trials (RCTs) that compared DOACs with warfarin, reporting on ischemic stroke, thromboembolism, and major hemorrhage as outcomes. Studies were excluded if they were observational, lacked relevant data, or did not meet the inclusion criteria specified and were extracted from PubMed, Cochrane Central, ClinicalTrials.gov, and Embase with the last search conducted in January 2025. The Cochrane Risk of Bias tool was used to assess the quality of the included studies. A meta-analysis was conducted using the inverse variance method with a fixed-effect model to pool OR with 95% CI for each outcome. Two studies with a total of 1115 participants were included. The DOAC group showed a significantly higher risk of ischemic stroke (OR: 17.42, P=0.005) and thromboembolism (OR: 3.58, P=0.001) compared to warfarin. No significant difference was found in the risk of major hemorrhage (OR: 0.89, P=0.70). The limitations of this review include the small number of included studies and the substantial heterogeneity in the thromboembolism outcomes (Chi²=0.03). There is also a potential for publication bias, as studies with negative or inconclusive results may be underrepresented. Additionally, inconsistencies in how thromboembolism and hemorrhagic events were defined across studies could have affected the outcome assessments. As a result, this meta-analysis implies that clinicians should exercise caution when prescribing DOACs to high-risk populations. Future studies, particularly larger, high-quality RCTs, are needed to further investigate these findings and to explore specific patient subgroups. No financial support was received for this review. This review was registered with PROSPERO (ID CRD42024625615) on December 12, 2024, prior to study search and data extraction.

## Introduction and background

Mechanical valve replacement (MVR) is a critical intervention for patients with severe valvular heart disease, necessitating lifelong anticoagulation to prevent thromboembolic events associated with mechanical prostheses [1]. Vitamin K antagonists (VKAs), such as warfarin, have been the standard anticoagulants for this purpose, demonstrating efficacy in reducing thromboembolic complications. However, their use is complicated by their narrow therapeutic index [2], which increases the risk of either thromboembolic events or major bleeding when the dosing is not carefully managed. Additionally, warfarin requires frequent monitoring through INR testing to ensure that patients remain within the therapeutic range, which can be both time-consuming and inconvenient for patients, while also being difficult for the healthcare provider to deliver. Beyond the monitoring, there are also numerous dietary restrictions, particularly with foods rich in vitamin K, which can interfere with the drug’s effectiveness making it difficult to maintain a stable therapeutic range and increasing the risk of either clotting or bleeding events [3]. Furthermore, warfarin carries a significant risk of severe bleeding [4], particularly in high-risk populations, such as those with mechanical heart valves or a history of thromboembolism. These challenges highlight the need for safer and more convenient anticoagulation options. Direct-acting oral anticoagulants (DOACs), including dabigatran, apixaban, rivaroxaban, and edoxaban, have transformed anticoagulation management in non-valvular atrial fibrillation (AF) and venous thromboembolism [5], offering more predictable pharmacokinetics, reduced monitoring needs, and lower bleeding risks. Despite these benefits, their use in patients with mechanical heart valves remains controversial. Given the limitations of VKAs and the risks of inadequate anticoagulation, it is essential to evaluate whether DOACs can provide a safer and more effective alternative for patients with MVR. This systematic review and meta-analysis aim to synthesize the available evidence on the efficacy and safety of DOACs in this population, examining their role in the reduction of thromboembolism and major bleeding. Specifically, it seeks to compare the efficacy of DOACs versus warfarin in preventing thromboembolic events and evaluate their safety profile concerning major bleeding and other adverse events. By addressing these objectives, the review aims to provide valuable insights for clinical decision-making and synthesize evidence used in the current clinical guidelines.

## Review

Methods

This systematic review and meta-analysis was performed in line with the Preferred Reporting Items for Systematic Reviews and Meta-Analysis (PRISMA) statement and recommendations of the Cochrane Collaboration Handbook for Systematic Reviews of Interventions [6]. The protocol of this meta-analysis was registered with PROSPERO on December 12, 2024 (ID CRD42024625615).

We systematically searched PubMed, Embase, Cochrane Central Register of Controlled Trials, and ClinicalTrials.gov databases between 01/01/2010 and 01/01/2025. The following terms were searched with Boolean operators: ((direct oral anticoagulant) OR (direct-acting oral anticoagulant) OR (DOAC) OR (Apixaban) OR (Betrixaban) OR (Edoxaban) OR (Rivaroxaban) OR (Dabigatran) OR (warfarin)) AND ((Mechanical aortic valve) OR (mechanical valve)) AND ((efficacy) OR (safety)). An English language restriction was applied to our search. Two authors (RB and NH) independently performed the literature search, blinded to each other’s work, following predefined search criteria. Any conflicts were resolved through discussion and consensus among the authors.

Studies were initially screened by reviewing their titles and abstracts. Those identified for inclusion were subsequently assessed through full-text screening.

Studies were included in the meta-analysis if they met the following criteria: enrolled participants were over the age of 18; the study population included individuals with MVR, with or without AF; outcomes were reported at least three months after MVR surgery; and the study was a randomized controlled trial (RCT).

Studies were excluded from the meta-analysis if they enrolled patients under the age of 18, if the study involved the enrolment of pregnant women, or if the studies were conducted prior to 2010. 

Two authors (RB and NH) independently reviewed each eligible study in full. The outcome data for thromboembolism, ischemic stroke, and major hemorrhage were systematically extracted and recorded in a standardized spreadsheet. In the case of discrepancies between the authors, these were discussed in detail until a consensus was reached. This process ensured the accuracy and consistency of the data included in the systematic review and meta-analysis.

The following data items were extracted from each included study: the total number of participants, the number of participants treated with each intervention, the drug intervention used, and the reported outcomes, including thromboembolism, ischemic stroke, and major hemorrhage. The study characteristics recorded encompassed details on study design, sample size, and follow-up duration. Intervention characteristics included the drug used and the duration of the intervention received by the participants.

The OR was used as the effect measure for the primary outcomes. A fixed-effect model was applied to pool the data across studies. The analysis was conducted with a 95% CI. The fixed-effect model assumes that the effect size is consistent across all studies and that any observed variation is due to sampling error.

No missing data was identified in any of the included studies, and data conversions were not required. To ensure data extraction consistency, two reviewers (RB and NH) independently extracted data while blinded to each other’s results. Extracted data was then compared and any discrepancies were discussed until a consensus was reached. No subgroup analysis was conducted.

Study selection was documented and visually displayed using a PRISMA flowchart. Intervention details and ORs were calculated and analyzed for each included study. The results were then presented as a forest plot, displaying effect sizes along with 95% CI for each individual outcome.

The results of individual studies were synthesized using meta-analysis to obtain a pooled effect estimate. A fixed-effect model was chosen for the meta-analysis based on the assumption that the true effect size is the same across all studies. This model was appropriate due to the homogeneity in study designs and participant characteristics.

To assess statistical heterogeneity, the I² statistic was calculated, which measures the percentage of total variation across studies due to heterogeneity. A Cochrane's Q test was performed to assess the presence of any heterogeneity, and Chi-squared tests were also used to test for overall effect size. If substantial heterogeneity was identified (I²>50%), a random-effects model would have been considered; however, RevMan 5.4 detected no significant heterogeneity in this analysis. Statistical significance was assessed using a Z-test with a significance threshold of P=0.005.

All statistical analyses were conducted using Cochrane’s RevMan Version 5.4 [7], which provided the tools for both conducting the meta-analysis and assessing heterogeneity. 

The Risk of Bias (RoB) in the included studies was assessed using the Cochrane RoB tool [8]. This tool evaluates studies across several domains, including selection bias, performance bias, detection bias, attrition bias, and reporting bias. The overall RoB for each study was determined based on the combined assessment across all domains. The results of the RoB assessment are summarized in Table 1. This assessment was used to inform the interpretation of the findings and to conduct sensitivity analyses, ensuring that studies with high RoB were appropriately considered in the synthesis.

**Table 1:** Risk of Bias Assessment Summary This table summarizes the Risk of Bias assessment for the RE-ALIGN trial [9] and the PROACT Xa trial [10].

	Selection	Performance	Detection	Attrition	Reporting
RE-ALIGN trial [9]	Low	High	Low	Unclear	Low
PROACT Xa trial [10]	Low	High	Low	Unclear	Unclear

Results

**Figure 1:**
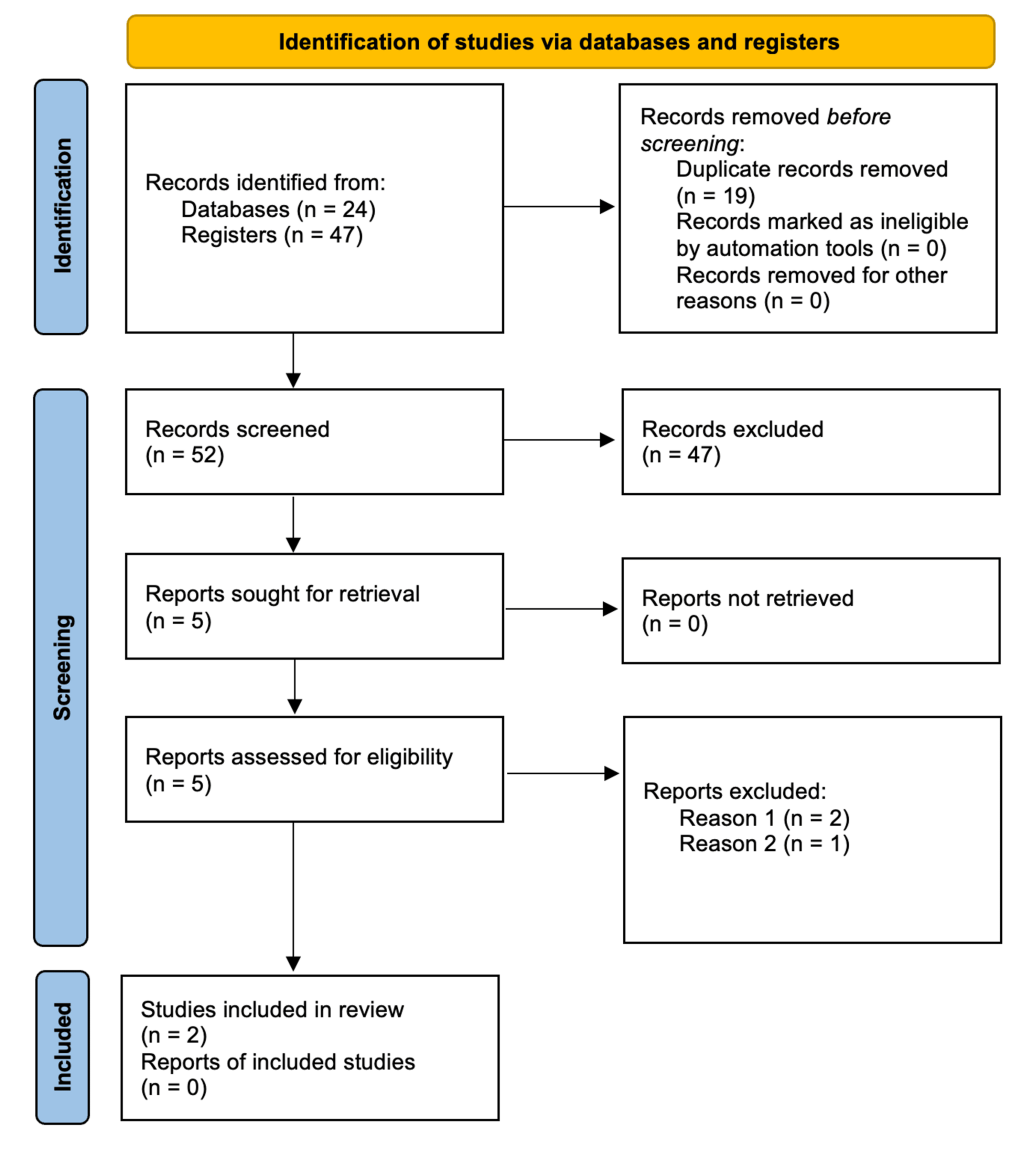
PRISMA Flowchart This flowchart was made in line with the PRISMA Statement [6] to demonstrate the inclusion and exclusion of studies identified from the database search.

A total of 71 records were identified from the search. After removing 18 duplicates, 53 records were screened by title and abstract. Of these, 47 records were excluded based on the screening criteria. Five records were then sought for retrieval, and all six were assessed for eligibility. This process is summarized in the PRISMA Flowchart (Figure 1). 

Several studies were initially identified as eligible based on their titles and abstracts but were excluded after full-text review. The reasons for exclusion of studies [11] and [12]: these studies were excluded as they did not include a direct comparison between DOACs and warfarin, focusing exclusively on one of these interventions without evaluating the relative effects of both. The reason for exclusion of study [13]: excluded due to its study design, as only RCTs were deemed eligible for inclusion in this review. 

The RE-ALIGN trial [9] was a prospective, randomized, phase 2, open-label study with blinded endpoint adjudication, conducted at 39 centers across 10 countries. Only data from the original 12-week phase of RE-ALIGN were included in our analysis, as the extension study data were not separately reported in sufficient detail. Eligible patients were aged 18-75 years and had undergone mechanical bileaflet valve implantation in the aortic and/or mitral position. The study included two populations: those who recently underwent valve implantation (Population A) and those with a mechanical mitral valve (with or without an aortic valve) implanted more than three months prior to randomization (Population B). Patients were randomly assigned in a 2:1 ratio to receive either dabigatran, with dose adjustments based on renal function to maintain a target plasma level of ≥50 ng/mL, or warfarin, with INR targets of 2.0-3.0 for low-risk patients and 2.5-3.5 for intermediate- or high-risk patients. The primary trial duration was 12 weeks, after which patients could transition to a long-term extension study (RE-ALIGN-EX) for up to 84 months. Efficacy outcomes included ischemic stroke, systemic embolism, transient ischemic attack, valve thrombosis, venous thromboembolism, myocardial infarction, and death, while safety outcomes focused on major bleeding as defined by the International Society on Thrombosis and Hemostasis (ISTH) criteria. Statistical analyses followed the intention-to-treat principle, with Cox proportional hazards models used for efficacy and safety assessments and a significance threshold of P≤0.05.

The PROACT Xa Trial [10] was a prospective, randomized, open-label study with blinded endpoint adjudication, designed by the steering committee and sponsor (Artivion) with the United States Food and Drug Administration (FDA) input. Institutional review boards approved the protocol and all participants provided written informed consent. Data was collected at study sites and analyzed at the Duke Clinical Research Institute. An independent data safety monitoring board reviewed unblinded data biannually, with additional unscheduled reviews as needed. Participants were enrolled at 64 U.S. sites and were eligible if they were ≥18 years old, had an On-X mechanical aortic valve implanted at least three months prior, and could receive warfarin (target INR 2.0-3.0). Patients were randomized 1:1 to receive either apixaban (5 mg twice daily, with dose reduction for select criteria) or warfarin, with monthly INR monitoring. Both groups had monthly follow-ups for adverse events and all participants reverted to warfarin at study termination. The primary efficacy endpoint was a composite of valve thrombosis or valve-related thromboembolism, adjudicated by a blinded clinical events committee. The primary safety endpoint was major bleeding, defined as any event leading to death, hospitalization, permanent injury, or requiring transfusion, pericardiocentesis, or reoperation. The sample size (990 participants) was based on an expected event rate of 1.75% per patient-year, with noninferiority established if the upper bound of the 95% CI was <1.75% per patient-year. Apixaban was compared to an FDA-defined objective performance criterion (OPC) of 1.6% per patient-year for thromboembolism and 0.1% for valve thrombosis. Cox proportional hazards models assessed efficacy and safety outcomes, with subgroup and sensitivity analyses conducted. Statistical analyses were performed using SAS version 9.4 at the Duke Clinical Research Institute.

The RoB in the RE-ALIGN study [9] was assessed across several domains. Selection bias was considered low, as the use of an automated randomization system suggests an appropriate process for random sequence generation, with a fixed allocation ratio that does not appear to introduce bias. The automated voice-response system for randomization further reduces the risk of allocation bias, although the absence of explicit mention of allocation concealment prevents a definitive assessment of this aspect. If the randomization system was functioning correctly, the risk of selection bias would remain low, but the open-label nature of the trial introduces the potential for investigator bias in outcome reporting. The risk of performance bias was judged to be high due to the lack of blinding in the study, as both participants and investigators knew the treatment assignment, which could influence participant behavior and treatment administration, leading to potential bias in outcomes. Detection bias was considered low, as the study employed blinded endpoint adjudication for primary and secondary outcomes, such as ischemic stroke, systemic embolism, and valve thrombosis. The risk of attrition bias was unclear, as the study did not provide detailed information regarding participant dropout rates or how missing data were handled, making it difficult to assess the full extent of this bias. Finally, the risk of reporting bias was deemed low, as the trial adhered to its protocol with respect to outcome reporting and the data presented were consistent with the pre-specified outcomes, suggesting minimal selective reporting.

The RoB in the PROACT Xa Trial [10] was evaluated across several domains. The risk of selection bias was considered low because participants were randomly assigned in a 1:1 ratio to receive either apixaban 5 mg twice daily or warfarin with a target INR of 2.0 to 3.0. Randomization was stratified based on whether the On-X valve was implanted less than or greater than one year before randomization. However, the lack of explicit detail of allocation concealment procedures, such as the use of sealed envelopes or a centralized randomization system, made the risk of allocation concealment unclear. The risk of performance bias was deemed high, as the trial was open-label, meaning that neither the participants nor the treating personnel were blinded to the assigned intervention. This lack of blinding could lead to differences in care, monitoring, or other interventions based on the treatment assignment. For instance, participants receiving apixaban may perceive it as a "newer" or "better" therapy, which could influence adherence or outcome reporting. The risk of detection bias was low, as the primary efficacy endpoint (valve thrombosis or valve-related thromboembolism) and the primary safety endpoint (major bleeding) were adjudicated by a blinded clinical events committee, reducing the risk of detection bias. The risk of attrition bias was unclear, as the trial was stopped early due to a higher rate of thromboembolic events in the apixaban group. While the decision to terminate the trial was made by an independent data safety monitoring board, the early termination could introduce attrition bias if it led to incomplete data collection or unequal follow-up times between groups. This risk was mitigated by standardized follow-up after trial termination and blinded adjudication of outcomes. The risk of reporting bias was also unclear. While the clear pre-specification of primary and secondary endpoints and independent data oversight reduce the likelihood of reporting bias, the early termination and lack of explicit mention of exploratory or non-significant results introduce the potential for incomplete reporting.

**Figure 2:**
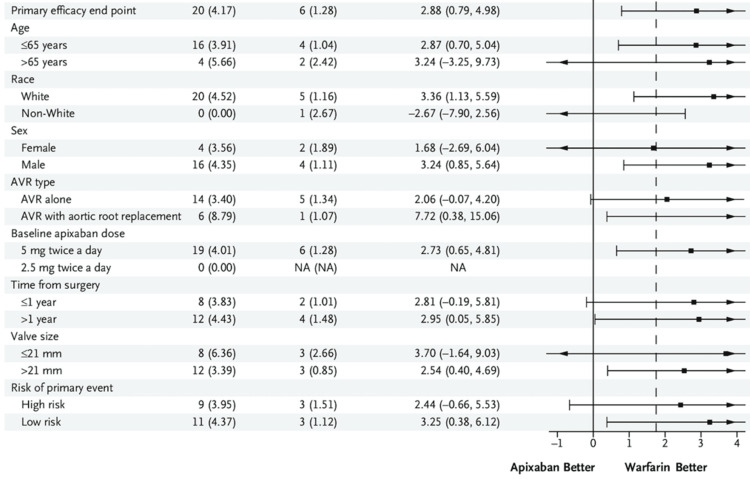
RE-ALIGN Trial Outcome Data Table This table summarizes the RE-ALIGN trial [9] outcome data. AVR, aortic valve replacement

In the RE-ALIGN trial [9], 433 patients were included in the dabigatran (DOAC) group and 430 were included in the warfarin group. In the dabigatran group, ischemic stroke occurred in nine patients and myocardial infarction in three patients, whereas no cases were reported in the warfarin group. Mortality was observed in one patient (<1%) in the dabigatran group and two patients in the warfarin group. Valve thrombosis without clinical symptoms occurred in five patients, all in the dabigatran group. The composite outcome of ischemic stroke, transient ischemic attack, systemic embolism, myocardial infarction, or death was reported in 15 patients in the dabigatran group and four patients in the warfarin group (HR: 1.94; 95% CI: 0.64-5.86; P=0.24). Major bleeding occurred in seven patients in the dabigatran group and two patients in the warfarin group, with any bleeding reported in 45 patients and 10 patients, respectively (HR: 2.45; 95% CI: 1.23-4.86; P=0.01). Most thromboembolic and major bleeding events were observed in patients randomized within one-week post-surgery (Population A). Findings in the as-treated population were consistent with the intention-to-treat analysis and no association was found between dabigatran plasma levels and thromboembolic or bleeding events. These outcomes are displayed in Figure 2. 

In the PROACT Xa Trial [10], 168 patients were included in the DOAC group and 84 patients were included in the warfarin group. In terms of efficacy, the primary composite end-point occurred in 20 events among 16 participants assigned to apixaban (4.2%/patient-year; 95% CI, 2.3 to 6.0) and six events in six participants assigned to warfarin (1.3%/patient-year; 95% CI, 0.3 to 2.3). The difference between groups was 2.9 percentage points per patient-year (95% CI, 0.8 to 5.0), which did not meet the margin for noninferiority. The observed event rate for apixaban (4.2%/patient-year) exceeded twice the OPC for valve thrombosis or valve-related thromboembolism (3.4%/patient-year). The observed event rates from the PROACT Xa trial (4.2% vs. 1.3% per patient-year) did not meet the predefined non-inferiority threshold (upper CI <1.75%). The hazard ratio for the primary efficacy outcome, valve thrombosis or valve-related thromboembolism, comparing apixaban to warfarin was 2.6 (95% CI, 1.0 to 6.7). Three valve thromboses and 14 ischemic strokes were observed in the apixaban group, while the warfarin group had five transient ischemic attacks and one myocardial infarction. The mortality rate was two deaths (apixaban) and one death (warfarin), all attributed to valve-related thromboembolism. Regarding the subgroupings of the PROACT Xa trial, the data was extracted directly from the trial's predefined analysis plan and published results.

For safety, there were 17 major bleeding events in 11 participants assigned to apixaban (3.6%/patient-year) and 21 events in 18 participants assigned to warfarin (4.5%/patient-year). Both groups exceeded twice the OPC for major bleeding (3.2%/patient-year). The hazard ratio for major bleeding comparing apixaban to warfarin was 0.6 (95% CI, 0.3 to 1.3). Intracranial hemorrhage occurred in one participant in the warfarin group and three participants in the apixaban group, all of whom also experienced an ischemic stroke. These outcomes are displayed in Table 2. However, the subgroup analyses presented in Table 2, particularly those comparing Populations A and B, are based on very low event counts and should be interpreted with caution. In several instances, hazard ratios were calculated from cells with zero to two events, resulting in wide confidence intervals (CIs) and limited reliability of the effect estimates. Therefore, these subgroup analyses should be interpreted as exploratory and having limited statistical power.

**Table 2:** PROACT Xa Trial Outcome Data Summary This table summarizes the PROACT Xa trial [10] outcome data.

Outcome	Population A		Population B		All Patients		Hazard Ratio (95% CI)	P-Value
	Dabigatran (N=133)	Warfarin (N=66)	Dabigatran (N=35)	Warfarin (N=18)	Dabigatran (N=168)	Warfarin (N=84)		
Death	1 (1)	2 (3)	0	0	1 (1)	2 (2)	0.25 (0.02-0.72)	0.26
Stroke	9 (7)	0	0	0	9 (5)	0	NA	NA
Transient Ischemic Attack	2 (2)	2 (3)	1 (3)	0	3 (2)	2 (2)	0.75 (0.13-4.49)	0.75
Bleeding (Major)	7 (5)	2 (3)	0	0	7 (4)	2 (2)	1.76 (0.37-8.45)	0.48

**Figure 3:**
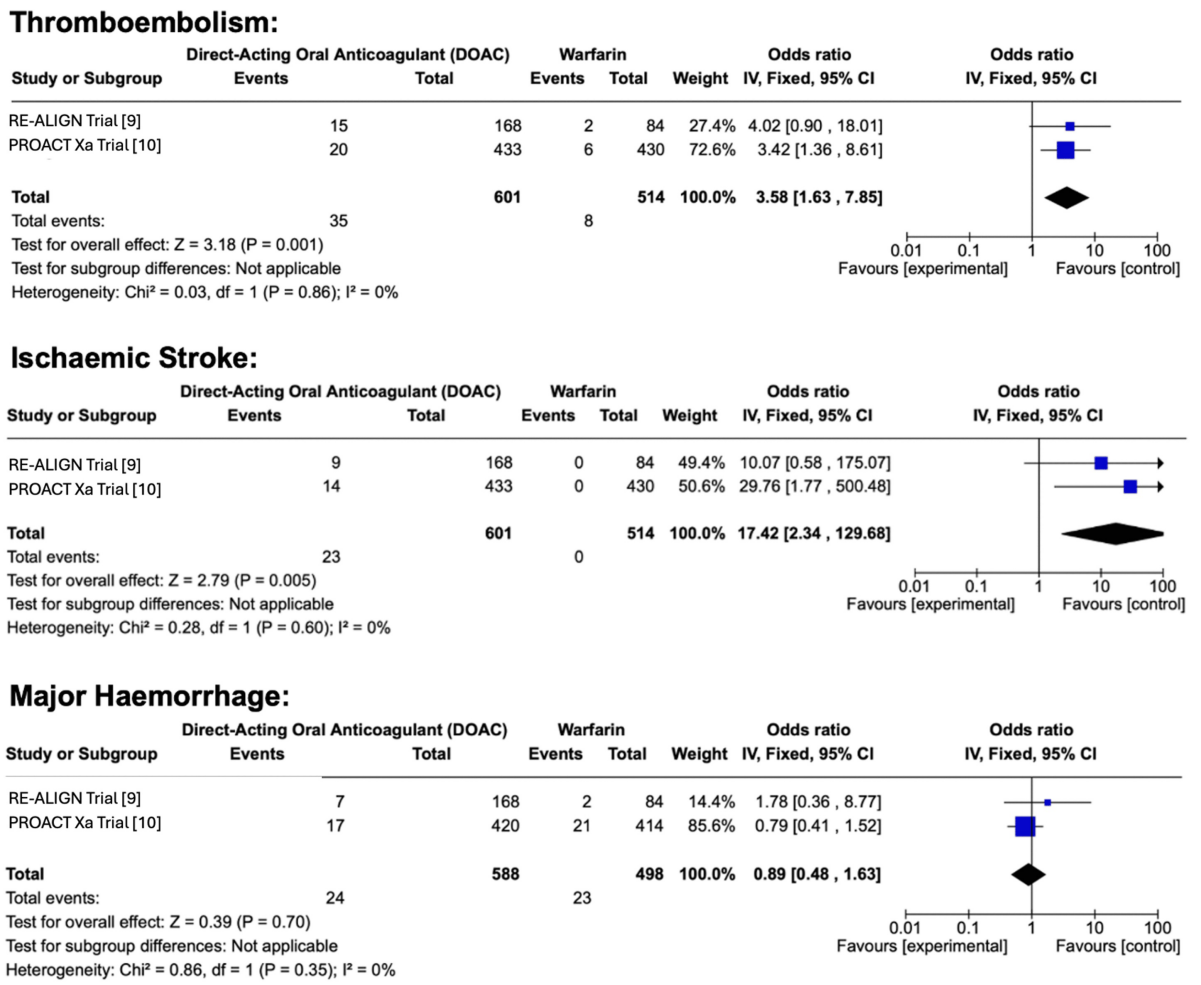
Diagram of Meta-Analysis Results for Each Outcome

In this meta-analysis comparing warfarin (control) to DOACs (experimental) for the outcome of ischemic stroke, two studies were included. The first study reported an OR of 10.07 with a weight of 49.4%, while the second study reported an OR of 29.76 with a weight of 50.6%. The pooled analysis yielded a combined OR of 17.42, indicating significantly increased odds of ischemic stroke in the experimental group compared to the control. The overall effect size was Z=2.79 (P=0.005), suggesting strong statistical significance. These findings imply that, in the included studies, the use of DOACs was observed to have a higher risk of ischemic stroke when compared to warfarin. For this outcome, there was no RoB due to missing results. A summary of these results displayed in the form of a forest plot can be seen in Figure 3. 

In this meta-analysis comparing warfarin (control) to DOACs (experimental) for the outcome of thromboembolism, two studies were included. The first study reported an OR of 4.02 with a weight of 27.4%, while the second study reported an OR of 3.42 with a weight of 72.6%. The pooled analysis yielded a combined OR of 3.58 (95% CI), indicating that the odds of thromboembolism were significantly higher in the experimental group compared to the control. The overall effect size was Z=3.18 (P=0.001), demonstrating strong statistical significance. These findings suggest that patients receiving DOACs were observed to have a significantly increased risk of thromboembolism compared to those receiving warfarin. For this outcome, there was no RoB due to missing results. A summary of these results displayed in the form of a forest plot can be seen in Figure 3. 

In this meta-analysis comparing warfarin (control) to DOACs (experimental) for the outcome of major hemorrhage, two studies were included. The first study reported an OR of 1.78 with a weight of 14.4%, while the second study reported an OR of 0.79 with a weight of 85.6%. The pooled analysis yielded a combined OR of 0.89 (95% CI), suggesting no significant difference in the risk of major hemorrhage between the two treatment groups. The overall effect size was Z=0.39 (P=0.70), indicating a lack of statistical significance. These findings imply that DOACs and warfarin were observed to have a comparable risk of major hemorrhage, with no clear advantage of one over the other in terms of bleeding complications. For this outcome, there was no RoB due to missing results. A summary of these results displayed in the form of a forest plot can be seen in Figure 3.

To assess statistical heterogeneity, a chi-squared test was performed for each outcome. For the outcome of ischemic stroke, the chi-squared test result was 0.28, indicating low heterogeneity between studies. For thromboembolism, the chi-squared test result was 0.03, suggesting the presence of significant heterogeneity. In contrast, for major hemorrhage, the chi-squared test result was 0.86, indicating minimal heterogeneity among the included studies.

The CIs for each outcome in this meta-analysis provide insight into the precision and reliability of the pooled effect estimates. For ischemic stroke, the combined OR was 17.42 with a 95% CI, indicating a significantly higher risk in the experimental group compared to the control. However, the wide CI suggests variability in the effect size, which may be influenced by differences in study populations or methodologies. For thromboembolism, the pooled OR was 3.58 with a 95% CI, demonstrating a significantly increased risk in the experimental group. The relatively narrower CI compared to ischemic stroke suggests more precision in the estimate, but the presence of statistical heterogeneity (Chi²=0.03) indicates potential variability across studies that should be explored further. For major hemorrhage, the pooled OR was 0.89 with a 95% CI, suggesting no significant difference in risk between the two treatment groups. The CI encompasses 1.0, indicating uncertainty regarding whether DOACs or warfarin are superior in terms of bleeding risk. Additionally, the high P-value (P=0.70) and low heterogeneity (Chi²=0.86) suggest that the effect estimate is stable, but further research may be needed to confirm this finding across diverse patient populations. Overall, while the CIs suggest strong associations between ischemic stroke and thromboembolism, the degree of certainty varies across outcomes due to differences in heterogeneity, sample sizes, and study methodologies. Further studies with larger sample sizes and more consistent methodologies may help refine these estimates and improve the precision of the findings. The disparity in outcomes between Population A and Population B can be seen in Figure 3. 

Discussion

Several limitations exist in the included evidence that may influence the interpretation of results. First, the number of studies included (n=2) in the meta-analysis was limited, reducing the generalizability of findings. The limited number of included studies means the results should be interpreted with caution and viewed as hypothesis-generating rather than definitive. Second, there was substantial heterogeneity in thromboembolism outcomes (Chi²=0.03), resulting from the following differences in stud methodology: PROACT Xa [10] evaluated apixaban in patients with mechanical aortic valves who were at least three months post-surgery and clinically stable, while RE-ALIGN [9] tested dabigatran in a broader population that included patients early after valve implantation and those with mitral valve prostheses, both higher-risk groups for thromboembolic events. Additionally, apixaban and dabigatran differ in their mechanisms of action and pharmacokinetics, with dabigatran showing variable absorption and a higher dependence on renal clearance, which may have contributed to subtherapeutic levels and poor outcomes in RE-ALIGN [9]. These design and drug-related differences underscore the importance of context when interpreting trial results and may help explain the contrasting findings regarding DOAC safety in patients with mechanical heart valves. 

Variability in patient populations, anticoagulant dosages, follow-up durations, and study designs may have contributed to this heterogeneity. Additionally, publication bias could not be ruled out, as studies with non-significant or unfavorable results may be underrepresented in the literature. The large disparity in event counts between treatment arms in the RE-ALIGN trial [9] (15 vs. 4 events) contrasts with a non-significant hazard ratio of 1.94 (95% CI: 0.64-5.86; P=0.24), which may appear counterintuitive to readers. This discrepancy is most plausibly explained by the small absolute number of outcome events and resulting low statistical power. With such few events, the CI is necessarily wide, reflecting considerable uncertainty around the point estimate. As a result, even a seemingly large observed difference in raw counts may not reach statistical significance. This underlines a broader issue in trials with low event rates: the inability to reliably detect true differences between treatment groups. In the context of this review, it reinforces the importance of considering both absolute event numbers and CIs when interpreting results, particularly when drawing comparisons across studies with differing event frequencies and durations of follow-up.

Although bleeding rates were numerically lower in the apixaban group, this apparent safety advantage must be weighed against the trial’s failure to demonstrate noninferiority for efficacy. Specifically, apixaban did not meet the predefined noninferiority margin for thromboembolic prevention, with a higher rate of ischemic stroke observed in the treatment arm. This underscores a critical trade-off in anticoagulation management for patients with mechanical heart valves: while minimizing bleeding risk is important, particularly in high-risk populations, it cannot come at the expense of adequate thromboembolic protection. The finding that all intracranial hemorrhages in the apixaban group were accompanied by ischemic strokes further complicates the interpretation, suggesting a possible overlap of thrombotic and hemorrhagic mechanisms or procedural factors. These results highlight the need for future trials to rigorously assess both efficacy and safety endpoints, ideally with adequate power to explore these outcomes separately and in combination. Clinically, the data support ongoing caution in the use of direct oral anticoagulants in this setting until more conclusive evidence becomes available. 

Finally, some included studies used varying definitions for thromboembolic and hemorrhagic events, which may have introduced inconsistencies in outcome assessment and comparability. For example, while one trial may have classified clinically silent events detected on imaging as thromboembolic events, another may have limited this category to symptomatic strokes or systemic embolism. Similarly, definitions of major bleeding differed across studies, with some adhering strictly to ISTH criteria, while others used broader or less clearly defined thresholds. These discrepancies complicate direct comparisons of event rates across studies and may contribute to heterogeneity in pooled estimates. In the context of this review, it is important to interpret outcome data with an understanding of these definitional differences, as they may have influenced both the reported incidence of adverse events and the overall conclusions about the safety profiles of the interventions being compared.

Socioeconomic and racial disparities may also partly explain the heterogeneity observed across the studies included in this meta-analysis. As highlighted by Borkowski et al. [14], individuals from marginalized backgrounds experience disproportionately higher rates of cardiovascular risk factors such as hypertension, diabetes, and obesity. These disparities are compounded by reduced access to healthcare, lower educational attainment, and increased psychosocial stressors, all of which may contribute to differential risks of thromboembolism, ischemic stroke, and major hemorrhage. While our review focused on clinical outcomes and did not stratify by socioeconomic status or ethnicity due to data limitations, the findings of Borkowski et al. [14] underscore the importance of considering these contextual factors when interpreting inter-study variability and generalizability. Future research should aim to incorporate more granular sociodemographic data to better understand how structural inequities shape cardiovascular outcomes and intervention effectiveness.

The systematic review and meta-analysis process itself has some inherent limitations. Although rigorous inclusion criteria were applied, potential selection bias cannot be excluded, particularly if relevant studies were missed due to database restrictions or unpublished data. Furthermore, reliance on study-reported data means that inconsistencies in outcome definitions or reporting biases could influence the pooled estimates. Another limitation is the inability to conduct more extensive subgroup analyses or meta-regression due to the limited number of studies, which restricts the ability to explore sources of heterogeneity in depth. Finally, while efforts were made to minimize bias through independent data extraction and conflict resolution, subjective judgment in study selection and data interpretation remains a potential limitation.

## Conclusions

The findings of this meta-analysis indicate that patients receiving DOACs had a significantly higher risk of ischemic stroke (OR: 17.42, P=0.005) and thromboembolism (OR: 3.58, P=0.001) compared to those receiving warfarin. However, no significant difference was observed in the risk of major hemorrhage between the two treatment groups (OR: 0.89, P=0.70). These results align with some previous studies suggesting that warfarin may offer superior thromboembolic protection in certain high-risk populations.

This meta-analysis has important implications for clinical practice and policy. Given the significantly higher odds of ischemic stroke and thromboembolism in the DOAC group compared to warfarin, clinicians should exercise caution when prescribing DOACs in high-risk patient populations. Future research should focus on conducting high-quality RTCs with larger sample sizes to confirm these findings. Further studies should ideally explore specific subgroups, such as elderly patients, those with renal impairment, and patients with varying thromboembolic risk profiles. This will help determine the most effective and safest anticoagulation strategies. Additionally, longer-term follow-up studies are needed to assess the real-world effectiveness and safety of DOACs compared to warfarin beyond the time frames of clinical trials.
